# The Role of Complement System in Septic Shock

**DOI:** 10.1155/2012/407324

**Published:** 2012-09-23

**Authors:** Jean Charchaflieh, Jiandong Wei, Georges Labaze, Yunfang Joan Hou, Benjamin Babarsh, Helen Stutz, Haekyung Lee, Samrat Worah, Ming Zhang

**Affiliations:** ^1^Department of Anesthesiology, SUNY Downstate Medical Center, 450 Clarkson Avenue, Brooklyn, NY 11203, USA; ^2^Department of Anesthesiology, Yale University School of Medicine, New Haven, CT 06510, USA; ^3^Department of Cell Biology, SUNY Downstate Medical Center, 450 Clarkson Avenue, Brooklyn, NY 11203, USA

## Abstract

Septic shock is a critical clinical condition with a high mortality rate. A better understanding of the underlying mechanisms is important to develop effective therapies. Basic and clinical studies suggest that activation of complements in the common cascade, for example, complement component 3 (C3) and C5, is involved in the development of septic shock. The involvement of three upstream complement pathways in septic shock is more complicated. Both the classical and alternative pathways appear to be activated in septic shock, but the alternative pathway may be activated earlier than the classical pathway. Activation of these two pathways is essential to clear endotoxin. Recent investigations have shed light on the role of lectin complement pathway in septic shock. Published reports suggest a protective role of mannose-binding lectin (MBL) against sepsis. Our preliminary study of MBL-associated serine protease-2 (MASP-2) in septic shock patients indicated that acute decrease of MASP-2 in the early phase of septic shock might correlate with in-hospital mortality. It is unknown whether excessive activation of these three upstream complement pathways may contribute to the detrimental effects in septic shock. This paper also discusses additional complement-related pathogenic mechanisms and intervention strategies for septic shock.

## 1. Introduction

Septic shock is a leading cause of morbidity and mortality among critically ill patients. Despite the use of potent antibiotics and improved intensive care, mortality rates of patients with severe sepsis and septic shock remain high (20–50%) [1–3]. A better understanding of the underlying mechanisms is important to develop future platforms of effective therapies.

Multiple mechanisms are likely involved in the development of septic shock. Host responses may initially respond to an infection but become amplified and dysregulated, resulting in hemodynamic collapse [4]. Decades of basic science and clinical research indicate that complement factors are involved in septic shock. While complement is an important defense system against bacterial infection, earlier clinical observations suggest that activation of complement factors is associated with detrimental effects in septic shock, such as multiorgan damages and poor outcome [5–8].

There are three pathways in the complement system: classical, alternative, and lectin. Different initiators activate each pathway but all converge to complement protein C3 and are followed by a common cascade (C5-9), resulting in the deposition of a membrane-attack-complex on targets and the release of chemoattractants (C3a and C5a) for inflammatory cells.

## 2. Pathophysiology of Complement**** Involvement in Septic Shock

### 2.1. Involvement of Complement Common Cascade in Septic Shock

A series of observations on C3 activation in septic shock patients were reported by a group of Dutch investigators led by Hack and Groeneveld. Activated C3 fragments, C3a and C3b/c, were elevated in septic shock patients and correlated with mortality [9–13]. Other clinical investigators also reported similar findings. Dofferhoff et al. found that, in 20 sepsis patients, C3a and C3d were elevated and that C3a levels correlated with Acute Physiology and Chronic Health Evaluation II (APACHE II) scores [14]. Furebring et al. showed that, in 12 patients with severe sepsis or septic shock, C3a (as well as C5b-9) levels were increased at the time of diagnosis [15]. These clinical observations suggest that C3 fragments released during septic shock may contribute to the development of fatal complications like profound hypotension and disseminated intravascular coagulation (DIC), thereby leading to a more severe disease course and a poor outcome.

It is interesting to note that some investigations did not conclude that C3 activation was detrimental in the development of severe sepsis. For instance, Shatney and Benner reported that in traumatic patients with acute systemic sepsis, serum C3 levels decreased shortly after admission [16]. Thereafter, C3 levels gradually returned to normal, despite the onset of fulminant systemic sepsis. These investigators argued that changes in C3 levels during severe sepsis were more consistent with protective host defense functions but did not support a role for C3 in the pathogenesis of acute fulminant clinical sepsis.

Basic science researchers have used various animal models to investigate the role of complement factors (mostly C3 and C5) in the common cascade. In a study using *E. coli* to induce septic shock in anaesthetized and artificially ventilated rabbits, circulating C5a positively correlated with endotoxin and the degree of accumulation of granulocytes in the lung tissue [17]. Using a baboon model with *E. coli*-induced septic shock, high amounts of endotoxin led to uncontrolled activation of complement C3 and C5-9 [18]. In a pig model of sepsis induced by fecal peritonitis, terminal complement complexeswere found to have been deposited in the kidneys [19].

The availabilities of several strains of complement factor knockout mice have greatly facilitated septic shock research, but conflicting results have been reported depending on the model that investigators chose. Yeh's group in Canada reported that like mice incapable of C3a/C3a receptor signaling, C5a receptor C5l2-deficient mice were hypersensitive to lipopolysaccharide- (LPS-) induced septic shock [20]. However, Ward's group in USA reported opposite findings using a mouse model of cecal ligation and puncture (CLP), which induces polymicrobial sepsis.In “mid-grade” sepsis (30–40% survival), blockade or absence of either C5a receptors, C5ar or C5l2, greatly improved survival and attenuated the buildup of proinflammatory mediators in plasma [21]. In “high-grade” sepsis (100% fatality), the only protective condition was the combined blockade of C5l2 and C5ar.Ward's group further showed that C5a induces apoptosis in adrenomedullary cells during experimental sepsis [22]. 

C3^−/−^ mice had significantly reduced survival in 2 septic shock models (LPS-induced and CLP) [23, 24]. Surprisingly, C5^−/−^ mice showed identical survival as wild type controls in CLP model [24]. In addition, C6-deficient rats had no significant differences in lung inflammation in an LPS-induced septic shock model [25].

These data suggest that C3 is essential to control bacteremia in 2 different models, but the functions of C5-9 seem dispensable. Depending on the type of animal model, anaphylatoxins C3a and C5a may be important factors in recruitment of inflammatory cells to combat infections but excessive release of these factors could be detrimental.

### 2.2. Involvement of Complement Classical Pathway in Septic Shock

Specific factors in complement classical pathway include immunoglobulins, C4 and C2. Animal studies showed that C3^−/−^, C4^−/−^, Btk^−/−^ (immunoglobulin deficient) and RAG-2^−/−^ (immunoglobulin deficient) mice were significantly more sensitive to endotoxin than wild-type controls [23, 26], suggesting an essential role for complement classical pathway to clear endotoxins.

Clinical investigators have explored the involvement of classical pathway in septic shock since the early 1980s. In a clinical study of 48 patients (19 with septic shock), those with septic shock had markedly decreased levels of C3, C4, and total complement activity as measured by the 50% hemolytic complement (CH_50_) assay. However, after 96 h, these values returned to baseline [27]. This underlines the transitory activation of the complement system through the classic pathway in septic shock. C4 activation, manifested as decrease of C4 or increase of C4a, was also reported in other clinical studies of severe late septic shock (42 patients) [28], clinically suspected sepsis on admission (43 of 47 patients) [13], severe sepsis and septic shock (50 patients) [29, 30]. IgG has also been associated with clinical outcomes of septic shock. In a study of 50 patients with severe sepsis or septic shock, survivors showed significantly higher levels of IgG upon diagnosis than those who ultimately died [30]. Thus, complement classical pathway is activated in septic shock as an essential response to clear endotoxins.

### 2.3. Involvement of Complement Alternative Pathway in Septic Shock

Specific factors in complement alternative pathway include factors B and D, which are regulated by factors H, I, and P (also called properdin). The alternative pathway is a critical defense system against bacterial infection. Patients with preexisting deficiency in factors of alternative pathway, for example, factor D and properdin, could suffer fulminant meningococcal infections resulting in septic shock [31–33].

Evidence of activation of alternative pathway in septic shock has been reported in a number of clinical studies. A study of 42 patients with severe late septic shock found factor B levels were significantly lower in patients who died than in patients who survived [28]. In these patients, the alternative pathway appears to be activated early in septic shock, whereas the classical pathway is activated later. Oglesby et al. also reported that among septic shock patients (both Gram-negative and Gram-positive) with preexisting cirrhosis, factor B levels were decreased [34]. Lin et al. reported that the active fragment of factor B, Bb as well as the Bb to factor B ratio, was significantly increased in septic shock patients [29]. Brandtzaeg et al. showed that among 20 patients with systemic meningococcal disease, ten patients with persistent septic shock had significantly higher levels of Bb than the other 10 patients without septic shock [35]. Finally, PBMCs from septic shock patients showed increased factor B mRNA expression when compared with control patients [36]. All these evidences suggest that complement alternative pathway is essential to fight against infections and is activated in clinical settings of septic shock.

### 2.4. Involvement of Complement Lectin Pathway in Septic Shock

Specific factors in complement lectin pathway include MBL and ficolin. Both MBL and ficolin circulate in complexes with one of three MBL-associated serine proteases (MASPs) [37, 38], with MASP-2 being the major complement-activating component among the three known MASPs. The MASP-2 is activated when MBL binds to certain carbohydrate or acetyl patterns on pathogens [39–41]. The activated MASP-2 then cleaves C4 and C2 to form the C3 convertase, C4b2a. MASP-2 activation is regulated by C1-inhibitor [42–44].

Basic research has shown that MBL and MASPs can bind to certain LPS containing a mannose homopolysaccharide. Such a binding of LPS to MBL and MASPs may cause C4 activation, resulting in the platelet response and development of rapid shock in mice [45].

However, two clinical studies from Eisen's group in Australia showed more complicated patterns of lectin complement activation in septic shock. In a study of 128 patients with sepsis and septic shock, the majority of patients did not display an MBL acute phase response on days 1, 3, 5, 7 [46]. Forty percent of these patients maintained consistent MBL levels throughout hospital stay, thirty percent of these patients had a positive acute phase response, and the remaining had a negative acute phase response [46]. In another study of 114 septic shock patients and 81 sepsis patients, MBL functional deficient patients had significantly higher sequential organ failure assessment (SOFA) scores, while higher MBL function and levels were found in patients who had SOFA scores predictive of good outcomes [47]. Thus, Eisen's group suggested that deficiency of MBL function may be associated with bloodstream infection and the development of septic shock.

Even with a likely protective role of MBL against sepsis, it is still possible that excessive activation of lectin pathway may contribute to the detrimental effects in septic shock. Sprong et al. reported two cases of MBL-deficient septic shock patients who had relatively low disease severity and mild disseminated intravascular coagulation (DIC) compared with 16 septic shock patients who had sufficient levels of MBL [48].

**Figure 1:**
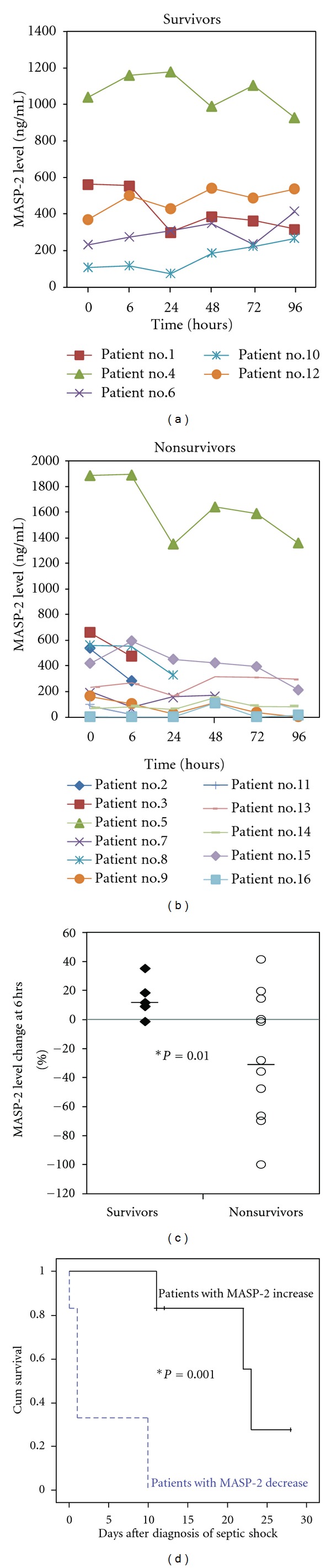
MASP-2 level changes in the survivors or nonsurvivors of septic shock. Plasma levels of MASP-2 were measured over the first 5 days in 16 patients after the diagnosis of septic shock. (a) MASP-2 profiles in the survivors: each line represents an individual patient; (b) MASP-2 profiles in the nonsurvivors; (c) comparison of the change of MASP-2 in survivors versus nonsurvivors at 6 hours after enrollment; (d) the association of changes in plasma levels of MASP-2 at 6 hrs after enrollment with hospital outcome. Kaplan-Meier survival analysis was performed to determine if patients with ≥10% increase of MASP-2 within 6 hours after the diagnosis of septic shock (solid line) had different in-hospital mortality rate compared with patients with ≥10% decrease of MASP-2 over the same time point (dashed line). *indicates statistical difference (*P* < 0.05).

It is still unclear whether other molecules in the lectin pathway are involved in septic shock. We have investigated the temporal patterns of MASP-2 in 16 septic shock patients and their correlation with in-hospital mortality. Our preliminary results showed that there was no difference in the baseline levels of MASP-2 between survivors and nonsurvivors. However, there was a trend that survivors had an increase of MASP-2 over the course of 5 days, while the nonsurvivors had a decrease during the same time period (Figures 1(a) and 1(b)). The change of MASP-2 in survivors at 6 hours after diagnosis of septic shock was significantly different than that of nonsurvivors (Figure 1(c)). Kaplan-Meier survival analysis showed that patients with ≥10% increase of MASP-2 within 6 hours after the diagnosis of septic shock had significantly less in-hospital mortality than patients with ≥10% decrease of MASP-2 during the same time period (Figure 1(d)). Therefore, acute decrease of MASP-2 during the early phase of septic shock may correlate with in-hospital mortality.

It remains to be determined to what degree is lectin complement activation necessary for protective effect against infection and whether there is threshold for the activation before detrimental effects appear. Future research, especially laboratory studies, may answer these questions.

### 2.5. Involvement of Other Complement-Related Inflammatory Mediators in Septic Shock

The development of septic shock is multifactorial and many potential mechanisms have been reviewed extensively by others [49–52]. Thus, this paper will only briefly describe the potential links between the complement system and its related inflammatory mediators in septic shock.

 Septic patients often exhibit a relative deficiency of C1-inhibitor (C1-INH) [53], which can inhibit activation of all 3 complement pathways [54–56]. C1-INH also inhibits proteases of the fibrinolytic, clotting, and kinin pathways. It is likely that during septic shock C1-INH may be depleted from the circulation by binding to factors in coagulation/fibrinolysis [57], thereby unable to control the excessive complement activation.

Cytokines and chemokines, particularly TNF-*α* and IL-6, are considered the first line biomarkers that drive the dynamic process of sepsis [58]. Cytokines and complement components can be activated similarly in sepsis [11, 14, 59–62] and their activation products may have overlapping biological activities [63]. Therefore, concomitant activation of cytokines and complements may amplify systemic inflammation leading to organ and system failure. 

Other circulating inflammatory mediators, including plasma prostaglandin (PGI) and phospholipase A-2 (PLA-2), may be activated in parallel with complements in septic shock and have direct association with complement factors [64, 65]. Thus, like cytokines, these mediators may have synergistic effects with complements in the development of septic shock.

## 3. Complement-Related Therapeutic Strategies**** for Septic Shock

Current management of septic shock includes early identification andtreatmentof the causative infection [66–68], adequate and rapid hemodynamic resuscitation [52, 69, 70], treatment of organ failure, corticosteroids [71], and modulation of the immune response [72, 73]. There are many comprehensive reviews on these topics and hence we will only review those strategies related to complements.

### 3.1. Strategies Directly Targeting Complement System

#### 3.1.1. Anti-C5a Treatment to Block Neutrophil Chemotaxis

Given the potential role of C5a in the development of septic shock, anti-C5a agents or C5a receptors-blockers may be reasonable therapeutic approaches. One benefit of C5a-blockade is that it still allows for terminal complement complex formation which is important to fight against infection. Anti-C5a has been tested in several septic shock models. In a primate model, anti-C5a antibody treatment significantly attenuated septic shock and pulmonary edema [74]. In a rat model of LPS-induced septic shock, pretreatment with F(ab′)2 fragments of rabbit anti-rat C5a did not change the circulating cell counts compared with LPS alone; however, a significant improvement in the mean arterial pressure and a decrease in hematocrit were observed [75]. In a pig model, pretreatment with anti-C5a mAb resulted in a decrease of serum IL-6 activity compared to control animals [76]. In a rodent model, blockade of C5a using neutralizing antibodies dramatically improved survival, reduced apoptosis of lymphoid cells, and attenuated the ensuing coagulopathy [77]. 

These anti-C5a approaches were based on the hypothesis (with supporting evidence) that C5a induced-neutrophil chemotaxis is harmful in septic shock. However, the effectiveness of anti-C5a in clinical settings remains to be tested. Even though it may have some beneficial effects, it may not be the ultimate solution for severe sepsis/shock, particularly because the role of neutrophils in sepsis may change at different stages of sepsis and inhibition of neutrophil chemotactic response could be detrimental in severe sepsis [78, 79]. 

#### 3.1.2. C1-INH

Administration of C1-INH can block complement activation, and its beneficial effect, although modest, in severe sepsis or septic shock has been demonstrated in animal studies [80, 81]. C1-INH has been tested in small clinical trials. In a trial of 7 patients with streptococcal toxic shock syndrome, administration of C1-INH markedly shifted fluid from extravascular to intravascular compartments, and 6 of these 7 patients survived [82]. In another trial of 40 patients with severe sepsis or septic shock, C1-INH treatment had a beneficial, although mild, effect on organ dysfunction [83]. These modest effects of C1-INH in septic shock are likely due to nonspecific inhibition of all 3 complement pathways. In another word, it not only suppresses complement-related systemic inflammation, but also hampers complement related antimicrobial responses.

### 3.2. Strategies Indirectly Affecting Complement System

Anti-TNF treatment (using humanized antibody to neutralize TNF-*α*) has been tested in a number of clinical trials of septic shock, and this topic has been thoroughly reviewed by others recently [84]. In short, although there was a trend of beneficial effect for anti-TNF to reduce sepsis-associated risk of death, none of these trials showed significance. One interesting notion is that inhibition of TNF alone does not reduce complement activation during sepsis, as demonstrated in a baboon model [85]. Thus, it is possible that inhibiting TNF without tackling excessive complement activation only provides a partial protection against systemic inflammation in septic shock. Combination of both anti-TNF and blockage of complement activation may lead to more effective protection.

Extracorporeal immunoadsorption (ECIA) using polymyxin B-immobilized fiber column hemoperfusion has been tested in animal models [86] and clinical trials [68, 87, 88]. ECIA has been used to adsorb endotoxin, monocytes, activated neutrophils, and anandamide [88], which may indirectly decrease complement activation [89, 90]. 

## 4. Conclusion

Complement system is essential to combat infections. However, it is a double-edged sword as excessive activation may cause severe injury to the host, as seen in septic shock. Direct or indirect inhibition of complement may provide new approaches in managing septic shock. Future research directions in this area may include (1) clarifying the pattern of complement activation, for example, time course; (2) determining the degree of involvement of three complement pathways in protection and detrimental effects; (3) reconciling the observations in different animal models; (4) further testing of anti-C5a and C1-INH in randomized clinical trials; (5) developing other specific and effective targeting strategies, for example, C3a inhibition.

## References

[1] Rangel-Frausto MS, Pittet D, Costigan M, Hwang T, Davis CS, Wenzel RP (1995). The natural history of the systemic inflammatory response syndrome (SIRS): a prospective study. *Journal of the American Medical Association*.

[2] Dombrovskiy VY, Martin AA, Sunderram J, Paz HL (2007). Rapid increase in hospitalization and mortality rates for severe sepsis in the United States: a trend analysis from 1993 to 2003. *Critical Care Medicine*.

[3] Martin GS, Mannino DM, Eaton S, Moss M (2003). The epidemiology of sepsis in the United States from 1979 through 2000. *The New England Journal of Medicine*.

[4] De Kock I, Van Daele C, Poelaert J (2010). Sepsis and septic shock: pathophysiological and cardiovascular background as basis for therapy. *Acta Clinica Belgica*.

[5] Robin M, Intrator L, Rapin M (1975). Letter: complement activation in septic shock. *The New England Journal of Medicine*.

[6] Graham DI, Behan PO, More IAR (1979). Brain damage complicating septic shock. Acute haemorrhagic leucoencephalitis as a complication of the generalised Shwartzman reaction. *Journal of Neurology Neurosurgery and Psychiatry*.

[7] Fritz H (1979). Proteinase inhibitors in severe inflammatory processes (septic shock and experimental endotoxaemia): biochemical, pathophysiological and therapeutic aspects. *Ciba Foundation Symposium*.

[8] Delsesto D, Opal SM (2011). Future perspectives on regulating pro-and anti-inflammatory responses in sepsis. *Contributions to Microbiology*.

[9] Hazelzet JA, de Groot R, van Mierlo G (1998). Complement activation in relation to capillary leakage in children with septic shock and purpura. *Infection and Immunity*.

[10] Groeneveld ABJ, Tacx AN, Bossink AWJ, van Mierlo GJ, Hack CE (2003). Circulating inflammatory mediators predict shock and mortality in febrile patients with microbial infection. *Clinical Immunology*.

[11] Groeneveld ABJ, Hack CE (2008). The role of the innate immune response in hospital- versus community-acquired infection in febrile medical patients. *International Journal of Infectious Diseases*.

[12] Hartemink KJ, Groeneveld ABJ (2010). The hemodynamics of human septic shock relate to circulating innate immunity factors. *Immunological Investigations*.

[13] Hack CE, Nuijens JH, Felt-Bersma RJF (1989). Elevated plasma levels of the anaphylatoxins C3a and C4a are associated with fatal outcome in sepsis. *American Journal of Medicine*.

[14] Dofferhoff ASM, de Jong HJ, Bom VJ (1992). Complement activation and the production of inflammatory mediators during the treatment of severe sepsis in humans. *Scandinavian Journal of Infectious Diseases*.

[15] Furebring M, Håkansson LD, Venge P, Nilsson B, Sjölin J (2002). Expression of the C5a receptor (CD88) on granulocytes and monocytes in patients with severe sepsis. *Critical Care*.

[16] Shatney CH, Benner C (1985). Sequential serum complement (C3) and immunoglobulin levels in shock/trauma patients developing acute fulminant systemic sepsis. *Circulatory Shock*.

[17] Bergh K, Olsen PO, Halgunset J, Iversen OJ (1991). Complement activation and pulmonary dysfunction in experimental *Escherichia coli* septicaemia. *Acta Anaesthesiologica Scandinavica*.

[18] Mollnes TE, Redl H, Hogasen K (1993). Complement activation in septic baboons detected by neoepitope-specific assays for C3b/iC3b/C3c, C5a and the terminal C5b-9 complement complex (TCC). *Clinical and Experimental Immunology*.

[19] Schuerholz T, Leuwer M, Cobas-Meyer M (2005). Terminal complement complex in septic shock with capillary leakage: marker of complement activation?. *European Journal of Anaesthesiology*.

[20] Chen NJ, Mirtsos C, Suh D (2007). C5L2 is critical for the biological activities of the anaphylatoxins C5a and C3a. *Nature*.

[21] Rittirsch D, Flierl MA, Nadeau BA (2008). Functional roles for C5a receptors in sepsis. *Nature Medicine*.

[22] Flierl MA, Rittirsch D, Chen AJ (2008). The complement anaphylatoxin C5a induces apoptosis in adrenomedullary cells during experimental sepsis. *PLoS ONE*.

[23] Fischer MB, Prodeus AP, Nicholson-Weller A (1997). Increased susceptibility to endotoxin shock in complement C3- and C4- deficient mice is corrected by C1 inhibitor replacement. *Journal of Immunology*.

[24] Flierl MA, Rittirsch D, Nadeau BA (2008). Functions of the complement components C3 and C5 during sepsis. *The FASEB Journal*.

[25] Brauer RB, Gegenfurtner C, Neumann B, Stadler M, Heidecke CD, Holzmann B (2000). Endotoxin-induced lung inflammation is independent of the complement membrane attack complex. *Infection and Immunity*.

[26] Reid RR, Prodeus AP, Khan W, Hsu T, Rosen FS, Carroll MC (1997). Endotoxin shock in antibody-deficient mice: unraveling the role of natural antibody and complement in the clearance of lipopolysaccharide. *Journal of Immunology*.

[27] León C, Rodrigo MJ, Tomasa A (1982). Complement activation in septic shock due to gram-negative and gram-positive bacteria. *Critical Care Medicine*.

[28] Sprung CL, Schultz DR, Marcial E (1986). Complement activation in septic shock patients. *Critical Care Medicine*.

[29] Lin RY, Astiz ME, Saxon JC, Saha DC, Rackow EC (1993). Alterations in C3, C4, factor B, and related metabolites in septic shock. *Clinical Immunology and Immunopathology*.

[30] Andaluz-Ojeda D, Iglesias V, Bobillo F (2011). Early natural killer cell counts in blood predict mortality in severe sepsis. *Critical Care*.

[31] Derkx HHF, Kuijper EJ, Fijen CAP, Jak M, Dankert J, Van Deventer SJH (1995). Inherited complement deficiency in children surviving fulminant meningococcal septic shock. *European Journal of Pediatrics*.

[32] Sprong T, Roos D, Weemaes C (2006). Deficient alternative complement pathway activation due to factor D deficiency by 2 novel mutations in the complement factor D gene in a family with meningococcal infections. *Blood*.

[33] Genel F, Atlihan F, Gulez N, Sjöholm AG, Skattum L, Truedsson L (2006). Properdin deficiency in a boy with fulminant meningococcal septic shock. *Acta Paediatrica*.

[34] Oglesby TJ, Schultz DR, Schein RMH, Sprung CL, Volanakis JE (1989). Measurement of complement proteins C2 and B in systemic lupus erythematosus and septic shock. *Complement and Inflammation*.

[35] Brandtzaeg P, Høgåsen K, Kierulf P, Mollnes TE (1996). The excessive complement activation in fulminant meningococcal septicemia is predominantly caused by alternative pathway activation. *Journal of Infectious Diseases*.

[36] Goring K, Huang Y, Mowat C (2009). Mechanisms of human complement factor B induction in sepsis and inhibition by activated protein C. *American Journal of Physiology*.

[37] Matsushita M, Fujita T (1992). Activation of the classical complement pathway by mannose-binding protein in association with a novel C1s-like serine protease. *Journal of Experimental Medicine*.

[38] Schwaeble W, Dahl MR, Thiel S, Stover C, Jensenius JC (2002). The mannan-binding lectin-associated serine proteases (MASPs) and MAp19: four components of the lectin pathway activation complex encoded by two genes. *Immunobiology*.

[39] Roos A, Bouwman LH, Munoz J (2003). Functional characterization of the lectin pathway of complement in human serum. *Molecular Immunology*.

[40] Turner MW (2003). The role of mannose-binding lectin in health and disease. *Molecular Immunology*.

[41] Tsutsumi A, Takahashi R, Sumida T (2005). Mannose binding lectin: genetics and autoimmune disease. *Autoimmunity Reviews*.

[42] Petersen SV, Thiel S, Jensen L, Vorup-Jensen T, Koch C, Jensenius JC (2001). Control of the classical and the MBL pathway of complement activation. *Molecular Immunology*.

[43] Presanis JS, Hajela K, Ambrus G, Gál P, Sim RB (2004). Differential substrate and inhibitor profiles for human MASP-1 and MASP-2. *Molecular Immunology*.

[44] Kerr FK, Thomas AR, Wijeyewickrema LC (2008). Elucidation of the substrate specificity of the MASP-2 protease of the lectin complement pathway and identification of the enzyme as a major physiological target of the serpin, C1-inhibitor. *Molecular Immunology*.

[45] Zhao L, Ohtaki Y, Yamaguchi K (2002). LPS-induced platelet response and rapid shock in mice: contribution of O-antigen region of LPS and involvement of the lectin pathway of the complement system. *Blood*.

[46] Dean MM, Minchinton RM, Heatley S, Eisen DP (2005). Mannose binding lectin acute phase activity in patients with severe infection. *Journal of Clinical Immunology*.

[47] Eisen DP, Dean MM, Thomas P (2006). Low mannose-binding lectin function is associated with sepsis in adult patients. *FEMS Immunology and Medical Microbiology*.

[48] Sprong T, Mollnes TE, Neeleman C (2009). Mannose-binding lectin is a critical factor in systemic complement activation during meningococcal septic shock. *Clinical Infectious Diseases*.

[49] Cinel I, Opal SM (2009). Molecular biology of inflammation and sepsis: a primer. *Critical Care Medicine*.

[50] Jean-Baptiste E (2007). Cellular mechanisms in sepsis. *Journal of Intensive Care Medicine*.

[51] Zanotti-Cavazzonia SL, Hollenberg SM (2009). Cardiac dysfunction in severe sepsis and septic shock. *Current Opinion in Critical Care*.

[52] Levy B, Collin S, Sennoun N (2010). Vascular hyporesponsiveness to vasopressors in septic shock: from bench to bedside. *Intensive Care Medicine*.

[53] Fourrier F, Jourdain M, Tournois A, Caron C, Goudemand J, Chopin C (1995). Coagulation inhibitor substitution during sepsis. *Intensive Care Medicine*.

[54] Davis AE (1989). Structure and function of C1 inhibitor. *Behring Institut Mitteilungen*.

[55] Matsushita M, Thiel S, Jensenius JC, Terai I, Fujita T (2000). Proteolytic activities of two types of mannose-binding lectin-associated serine protease. *Journal of Immunology*.

[56] Jiang H, Wagner E, Zhang H, Frank MM (2001). Complement 1 inhibitor is a regulator of the alternative complement pathway. *Journal of Experimental Medicine*.

[57] Wuillemin WA, Fijnvandraat K, Derkx BHF (1995). Activation of the intrinsic pathway of coagulation in children with meningococcal septic shock. *Thrombosis and Haemostasis*.

[58] Gullo A, Foti A, Murabito P (2010). Spectrum of sepsis, mediators, source control and management of bundles. *Frontiers in Bioscience*.

[59] de Boer JP, Wolbink GJ, Thijs LG, Baars JW, Wagstaff J, Hack CE (1992). Interplay of complement and cytokines in the pathogenesis of septic shock. *Immunopharmacology*.

[60] Hack CE, De Groot ER, Felt-Bersma RJF (1989). Increased plasma levels of interleukin-6 in sepsis. *Blood*.

[61] Brandtzaeg P, Osnes L, Ovstebo R, Joo GB, Westvik AB, Kierulf P (1996). Net inflammatory capacity of human septic shock plasma evaluated by a monocyte-based target cell assay: identification of interleukin-10 as a major functional deactivator of human monocytes. *Journal of Experimental Medicine*.

[62] Bossink AWJ, Paemen L, Jansen PM, Hack CE, Thijs LG, Van Damme J (1995). Plasma levels of the chemokines monocyte chemotactic proteins-1 and -2 are elevated in human sepsis. *Blood*.

[63] Wakabayashi G, Gelfand JA, Jung WK, Connolly RJ, Burke JF, Dinarello CA (1991). Staphylococcus epidermidis induces complement activation, tumor necrosis factor and interleukin-1, a shock-like state and tissue injury in rabbits without endotoxemia: comparison to *Escherichia coli*. *Journal of Clinical Investigation*.

[64] Slotman GJ, Burchard KW, Williams JJ (1986). Interaction of prostaglandins, activated complement, and granulocytes in clinical sepsis and hypotension. *Surgery*.

[65] Gijon MA, Perez C, Mendez E, Sanchez Crespo M (1995). Phospholipase A2 from plasma of patients with septic shock is associated with high-density lipoproteins and C3 anaphylatoxin: some implications for its functional role. *Biochemical Journal*.

[66] Funk D, Sebat F, Kumar A (2009). A systems approach to the early recognition and rapid administration of best practice therapy in sepsis and septic shock. *Current Opinion in Critical Care*.

[67] Textoris J, Wiramus S, Martin C, Leone M (2011). Antibiotic therapy in patients with septic shock. *European Journal of Anaesthesiology*.

[68] Davies B, Cohen J (2011). Endotoxin removal devices for the treatment of sepsis and septic shock. *The Lancet Infectious Diseases*.

[69] Ramos FJD, Azevedo LC (2010). Hemodynamic and perfusion end points for volemic resuscitation in sepsis. *Shock*.

[70] Rivers EP, Jaehne AK, Eichhorn-Wharry L, Brown S, Amponsah D (2010). Fluid therapy in septic shock. *Current Opinion in Critical Care*.

[71] Annane D, Bellissant E, Bollaert PE (2009). Corticosteroids in the treatment of severe sepsis and septic shock in adults: a systematic review. *Journal of the American Medical Association*.

[72] Vincent JL, Carrasco Serrano E, Dimoula A (2011). Current management of sepsis in critically ill adult patients. *Expert Review of Anti-Infective Therapy*.

[73] Ramakers BP, Riksen NP, van der Hoeven JG, Smits P, Pickkers P (2011). Modulation of innate immunity by adenosine receptor stimulation. *Shock*.

[74] Hangen DH, Stevens JH, Satoh PS, Hall EW, O’Hanley PT, Raffin TA (1989). Complement levels in septic primates treated with anti-C5a antibodies. *Journal of Surgical Research*.

[75] Smedegard G, Cui L, Hugli TE (1989). Endotoxin-induced shock in the rat. A role for C5a. *American Journal of Pathology*.

[76] Höpken U, Mohr M, Strüber A (1996). Inhibition of interleukin-6 synthesis in an animal model of septic shock by anti-C5a monoclonal antibodies. *European Journal of Immunology*.

[77] Ward PA, Guo RF, Riedemann NC (2012). Manipulation of the complement system for benefit in sepsis. *Critical Care Research and Practice*.

[78] Alves-Filho JC, Spiller F, Cunha FQ (2010). Neutrophil paralysis in sepsis. *Shock*.

[79] Reddy RC, Standiford TJ (2010). Effects of sepsis on neutrophil chemotaxis. *Current Opinion in Hematology*.

[80] Jansen PM, Eisele B, de Jong IW (1998). Effect of C1 inhibitor on inflammatory and physiologic response patterns in primates suffering from lethal septic shock. *Journal of Immunology*.

[81] Zhang H, Li J, Barrington RA, Liang G, Qin G, Liu DX (2007). An anti-endotoxin peptide that generates from the amino-terminal domain of complement regulatory protein C1 inhibitor. *Biochemical and Biophysical Research Communications*.

[82] Fronhoffs S, Luyken J, Steuer K, Hansis M, Vetter H, Walger P (2000). The effect of C1-esterase inhibitor in definite and suspected streptococcal toxic shock syndrome. Report of seven patients. *Intensive Care Medicine*.

[83] Zeerleder S, Caliezi C, van Mierlo G (2003). Administration of C1 inhibitor reduces neutrophil activation in patients with sepsis. *Clinical and Diagnostic Laboratory Immunology*.

[84] Qiu P, Cui X, Barochia A, Li Y, Natanson C, Eichacker PQ (2011). The evolving experience with therapeutic TNF inhibition in sepsis: considering the potential influence of risk of death. *Expert Opinion on Investigational Drugs*.

[85] Bengtsson A, Redl H, Schlag G, Högåsen K, Götze O, Mollnes TE (1998). Anti-TNF treatment of baboons with sepsis reduces TNF-*α*, IL-16 and IL- 8, but not the degree of complement activation. *Scandinavian Journal of Immunology*.

[86] Sato T, Orlowski JP, Zborowski M (1993). Experimental study of extracorporeal perfusion for septic shock. *American Society for Artificial Internal Organs Journal*.

[87] Stegmayr BG (1996). Plasmapheresis in severe sepsis or septic shock. *Blood Purification*.

[88] Mitaka C, Tomita M (2011). Polymyxin B-immobilized fiber column hemoperfusion therapy for septic shock. *Shock*.

[89] Reeves JH, Butt WW, Shann F (1999). Continuous plasmafiltration in sepsis syndrome. *Critical Care Medicine*.

[90] Schefold JC, von Haehling S, Corsepius M (2007). A novel selective extracorporeal intervention in sepsis: immunoadsorption of endotoxin, interleukin 6, and complement-activating product 5a. *Shock*.

